# Dr. Joseph R. Laine

**Published:** 1903-02

**Authors:** 


Dr. Joseph R. Laine, of San Francisco, U. of B. '76, died at the German Hospital in the city of his home, December 15, 1902, aged 56 years. He had been ill for a long time, suffering from cardiac disease. Dr. Laine settled first in Peoria, Ill., then removed to Nebraska, and finally to California, locating first at Sacramento and afterward at San Francisco. During his residence in California he served for eight years as a member of the State Board of Health, a portion of the time being secretary. He was one of the founders of the College of Physicians and Surgeons in San Francisco, of which he was president during several years. He was also Assistant Surgeon-General of the California National Guard, holding the rank of lieutenant-colonel.

